# Percutaneous microchannel unilateral approach bilateral micro decompression for adjacent segmental degeneration after lumbar fusion at 10 years: a case report and review of literature

**DOI:** 10.3389/fsurg.2024.1284967

**Published:** 2024-01-24

**Authors:** Tingxin Zhang, Gang Gao, Feng Gao, Nana Guo, Yongjiang Wang

**Affiliations:** ^1^ Department of Orthopedics, Ordos Central Hospital, Ordos, China; ^2^ Critical Care Medicine, Ordos Central Hospital, Ordos, China

**Keywords:** percutaneous microchannel, adjacent segmental degeneration, microchannel, micro decompression, lumbar

## Abstract

**Background:**

Adjacent segmental degeneration after lumbar fusion is one of the common long-term complications after lumbar fusion. With the continuous development of adjacent segmental degeneration, patients who fail conservative treatment often need reoperation to relieve symptoms. In recent years, the technique of bilateral microdecompression through unilateral approach under microchannel has been widely used in the treatment of lumbar degenerative diseases. However, the efficacy of this procedure for adjacent-segment degeneration after lumbar fusion has not been established. Here, we report a case of bilateral microscopic decompression via a unilateral approach through a microchannel in a patient with adjacent segmental degeneration after lumbar fusion.

**Case report:**

A 70-year-old male patient was admitted to hospital because of lumbago accompanied by left lower extremity pain, numbness and weakness for 2 years, which aggravated for 2 months. Ten years ago, he underwent PLIF for lumbar spinal stenosis, and recovered well after the operation. According to imaging data and physical examination, the diagnosis was adjacent segmental degeneration after lumbar fusion. Bilateral microdecompression was performed through a unilateral approach under a microchannel. Good clinical outcomes was observed through 1-year postoperative follow-up.

**Conclusions:**

This report reports the successful treatment of a patient with ASD 10 years after lumbar fusion. Bilateral microdecompression via a unilateral approach under a microchannel is a safe and effective method for the treatment of ASD after lumbar fusion with good surgical outcomes.

## Introduction

Degenerative lumbar disease can be treated with lumbar fusion, but it may lead to spinal lesions in previously fused levels (1, 2). Adjacent segment degeneration (ASD) is a long-term complication that can occur after lumbar fusion, which refers to the degeneration of the intervertebral disc, instability, and slippage in the adjacent segment of the fusion segment (3). Current retrospective studies have shown that lumbar fusion surgery may lead to problems with adjacent motion segments, specifically post-fusion ASD (4). Battie et al. (5) found that fusion surgery does not directly cause this phenomenon, ASD can be considered a natural part of the aging process. However, changes in stress may play a role in this process. An *in vitro* mechanical study found that lumbar fusion can cause abnormal pressure in the intervertebral disc and excessive motion of adjacent spinal segments, which may contribute to ASD (6). As ASD continues to evolve, patients who do not respond to conservative treatment may require reoperation to alleviate symptoms. Numerous studies have indicated that adjacent segment degeneration occurs in 5%–75% of cases after lumbar fusion, with 20% requiring additional surgical intervention (7, 8). As ASD continues to develop, patients who do not respond to conservative treatment often require reoperation to alleviate symptoms. One common approach to addressing ASD after lumbar fusion is through posterior lumbar interbody fusion (PLIF). However, revising ASD with traditional PLIF carries certain risks. This operation involves extensive soft tissue stripping to expose the original internal fixation, resulting in significant bleeding, high postoperative pain levels at the incision site, a long recovery time, and high costs. In recent years, surgeons have been exploring less invasive procedures in order to enhance clinical outcomes. One such procedure, proposed by Young et al. (9) was the use of microscope-assisted unilateral laminectomy for the treatment of lumbar spinal stenosis. This procedure involves peeling off the muscles on one side of the paravertebral side and performing microdecompression delicately under a microscope. In recent years, minimally invasive spinal surgeries such as unilateral approach and bilateral microdecompression under percutaneous microchannel have become the forefront of spinal treatment. The intermuscular approach is used instead of the traditional subperiosteal muscle stripping, which reduces tissue damage and speeds up recovery. However, the effectiveness of bilateral microdecompression via a unilateral approach through percutaneous microchannels in treating ASD after lumbar fusion has not been established. This case report presents a successful treatment of ASD 10 years after lumbar fusion using bilateral microdecompression through a unilateral approach via percutaneous microchannel.

### Case presentation

A 70-year-old male patient was admitted to hospital because of lumbago accompanied by left lower extremity pain, numbness and weakness for 2 years, which aggravated for 2 months. Ten years ago, he underwent PLIF for lumbar spinal stenosis, and recovered well after the operation. Physical examination revealed tenderness and percussion pain in the L3–4 spine area, and limited lumbar extension. Decreased skin sensation in the medial calf and medial malleolus of the left lower extremity. The muscle strength of the left lower extremity was grade III, the muscle strength of the right lower limb was grade IV, and the muscle tone was normal. The straight leg raising test of the left lower extremity was positive (30 degrees), and the straight leg raising test of the right lower extremity was negative. Physiological reflexes of the lower limbs were elicited normally, but pathological reflexes were not elicited. Based on the Japanese Orthopaedic Association (JOA) scoring system, the neurological function score of the patient was 10 points. Back pain Visual Analogue Scale (VAS) score is 7 points, leg pain VAS score is 8 points. Preoperative lumbar spine Oswestry Disability Index (ODI) score was 60%.

**Figure 1:**
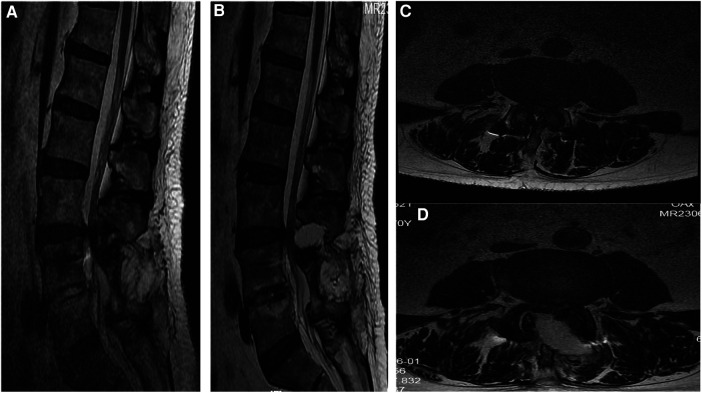
Preoperative and postoperative thoracic MRI. (**A**) Sagittal T2-weighted imaging showed spinal canal stenosis at the L3/4. (**B**) Three months after the operation, the sagittal view of the lumbar spine MRI showed satisfactory decompression at L3/4. (**C**) Axial t2-weighted imaging showing hypertrophy of the ligamentum flavum at the L3/4 level, causing spinal stenosis. (**D**) Three months after the operation, the lumbar spine MRI axial view showed satisfactory decompression at L3/4.

Magnetic resonance imaging (MRI) revealed disc herniation at the L3/4 level, along with hypertrophy of the ligamentum flavum, inward hyperplasia of the articular process, and spinal canal stenosis (Figure 1). A diagnosis of ASD after lumbar fusion was made. The treatment plan for ASD after lumbar fusion involved bilateral microdecompression through a unilateral percutaneous microchannel approach.

### Surgical treatment

**Figure 2:**
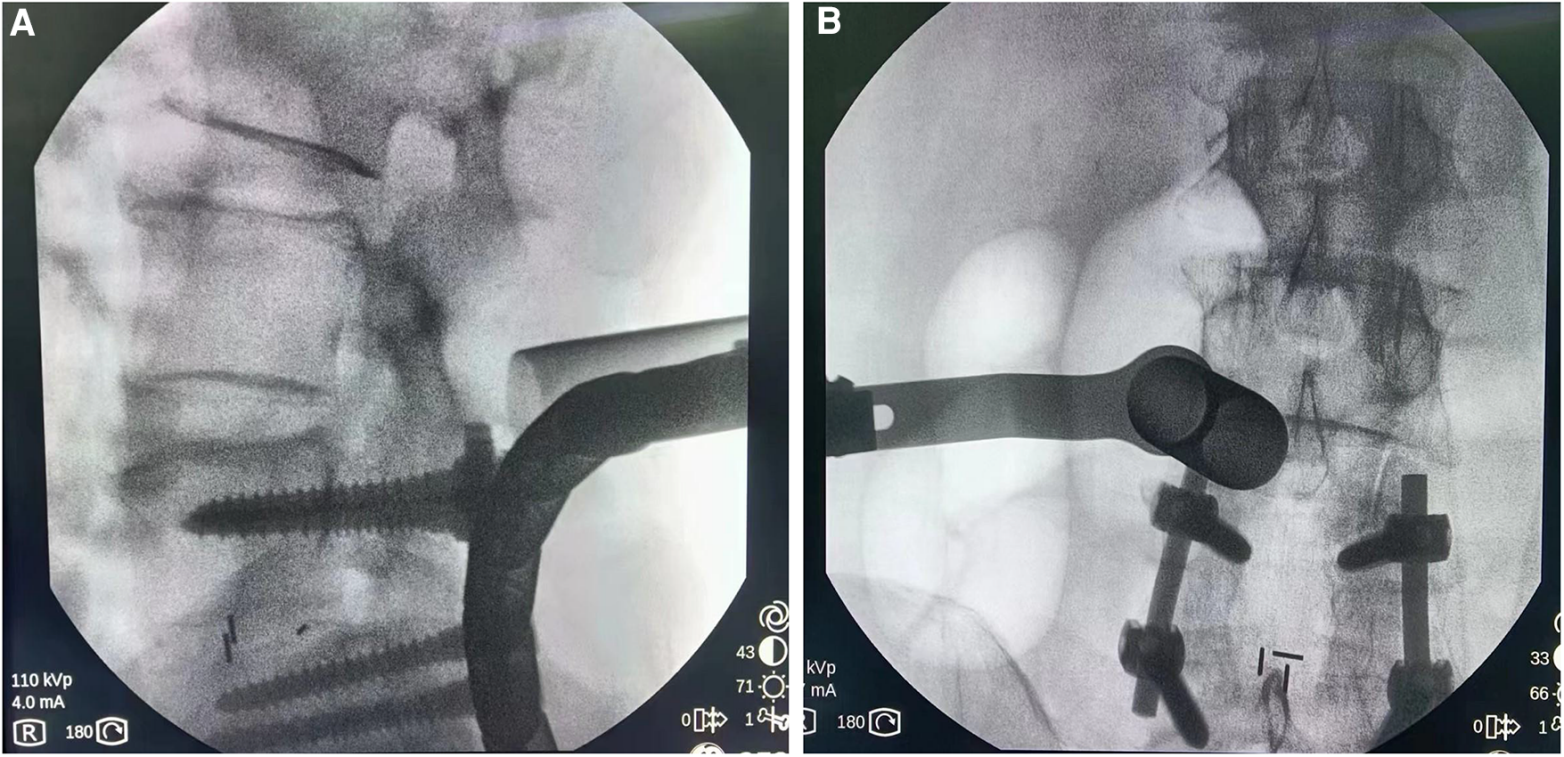
Intraoperative fluoroscopy (**A**) lateral view shows channel localization at L3/4 level. (**B**) The orthographic map shows channel localization at the L3/4 level.

**Figure 3:**
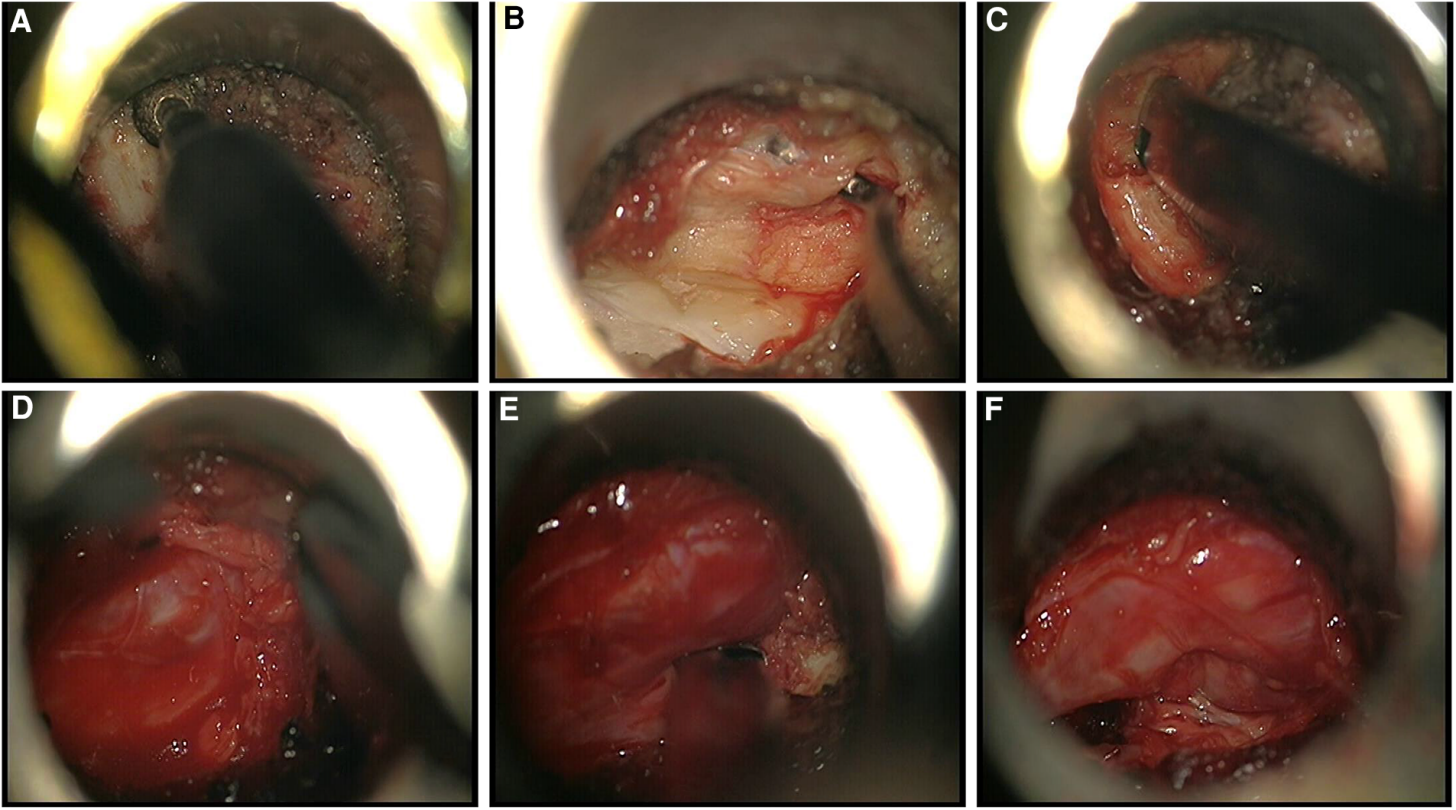
Intraoperative findings. (**A**) Removal of the lamina and spinous process bases using a powered system. (**B)** Exposed hypertrophic ligamentum flavum. (**C**) Removal of hypertrophic ligamentum flavum using Kerrison rongeurs. (**D**) Bipolar radiofrequency was used for hemostasis. (**E**) Removal of hypertrophic bone tissue using Kerrison rongeurs. (**F**) Nerve root after decompression.

After successful general anesthesia, the patient was placed in a prone position with the abdomen suspended, and a neurophysiological monitoring system was established. Accurately locate the narrowed spinal canal under the C-arm, and make a longitudinal skin incision 2.5 cm to the left of the posterior midline at the position corresponding to the spinal canal stenosis, with a length of about 1.8 cm. The subcutaneous and fascia were incised sequentially, and the paravertebral expansion cannula was used to bluntly separate the muscle layer step by step through a Gram needle, and a surgical microchannel (diameter of 1.6 cm) was inserted under the guidance of the expansion cannula (Figure 2). The expansion sleeve was placed along the periphery and connected to the fixation rod through a serpentine chain. It was then fixed beside the bed. The C-arm was used to confirm that the channel was at the level of the spinal canal stenosis. Install a microscope, use a drill to remove the root of the lumbar 3 spinous process and the lower edge of the L3 lamina, and open the lamina according to the preoperative imaging data. The ligamentum flavum is then removed with a rongeur. The left lumbar 4 nerve root was released, the nerve root canal was enlarged, and it was observed that the nerve was obviously released. Adjust the direction of the working channel, grind the right part of the inferior articular process of L3, resect the thickened ligamentum flavum between L3 and L4 on the right side, loosen the nerve root of L4 on the right side, expand the nerve root canal, and observe that the nerve is obviously released2 (Figure 3). During the surgery, the amount of blood lost was approximately 30 ml. Following the procedure, there was a slight improvement in muscle strength in the lower extremities.

## Results

**Figure 4:**
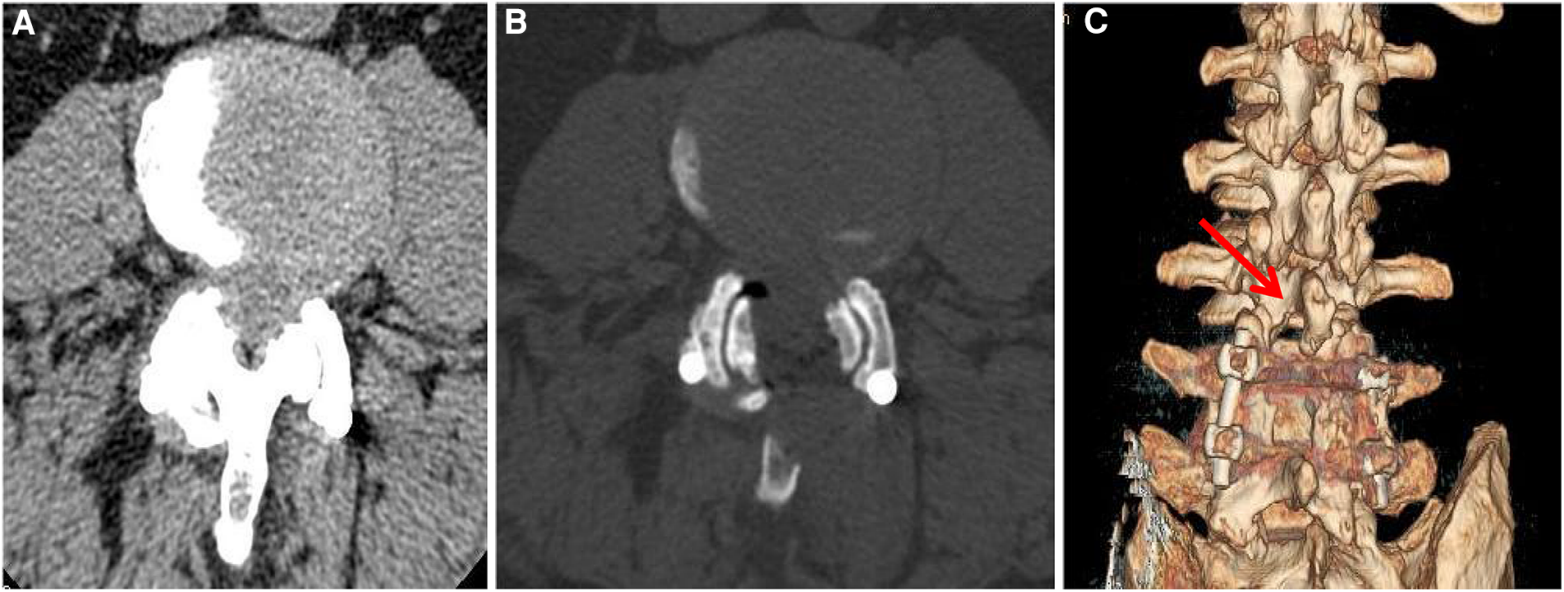
Preoperative and postoperative computed tomography (CT). (**A**) Preoperative CT showed hypertrophy of the ligamentum flavum at the L3/4 level, hypertrophy of the facet joints, and spinal canal stenosis. **(B**) Three months after the operation, CT of the lumbar spine showed satisfactory decompression at the L3/4 level, and the facet joints were well preserved. (**C**) At 3 months after the operation, three-dimensional reconstruction CT of the lumbar spine showed that the facet joints at the L3/4 level were well preserved.

The patient experienced immediate relief from lower extremity and back pain after the operation. At last follow-up, the patient's VAS score for back pain and leg pain was 2. Within 2 weeks, the muscle strength of the left lower limb improved to grade IV, and the muscle strength of the right lower limb improved to grade IV+. Prior to the operation, the patient had a JOA score of 10 points. After 1 year of follow-up, the JOA score increased to 23 points, resulting in a 73% improvement rate in JOA score after treatment. The patient had an ODI score of 60% prior to the operation. After a year of follow-up, the ODI score showed significant improvement, reaching 10% (Table 1). Postoperative CT and MRI showed satisfactory decompression at the L3–4 level, the articular process was well preserved, and the stability of the lumbar spine was not damaged (Figures 1, 4).

**Table 1:** Patient follow-up results.

	Preoperative	Postoperative			
		1 week	1 month	3 months	12 months
Back pain VAS	7	4	3	2	2
Leg pain VAS	8	4	2	2	2
ODI	60	31	16	12	10
JOA	10	15	19	22	24

VAS, visual analog score (0–10); ODI, Oswestry disability index (0%–100%); JOA, Japanese Orthopaedic Association (0–29).

## Discussion

This study reports on the clinical effects of 10-year ASD after lumbar fusion with bilateral microdecompression via a unilateral approach under a microchannel. The method used in this study can effectively decompress neural structures without significantly destabilizing the spine. The results showed significant improvement in VAS scores for back pain and leg pain, with a JOA improvement rate of 73% at the last follow-up. Postoperative imaging results indicated that there was no obvious spinal instability at the decompression segment and the decompression effect was satisfactory. Lumbar fusion is a well-established treatment option for degenerative lumbar disease (10, 11). When adjacent segment disease (ASD) develops following lumbar fusion, many surgeons opt for decompression by extending internal fixation (12, 13). However, we propose a more minimally invasive approach that involves bilateral microdecompression through a unilateral approach under a microchannel. This approach has yielded good surgical outcomes for patients with ASD after lumbar fusion. Our results are comparable or even better than those reported in other studies that have used extended decompression with internal fixation after lumbar fusion.

When considering treatment options for the lumbar spine, the cost of hospitalization is an important factor for patients. Bilateral microdecompression via a unilateral approach under a microchannel is a less expensive option compared to extended internal fixation and fusion because it does not require implant costs (14). Lumbar fusion surgery has been found to have a higher incidence of perioperative morbidity, as well as longer hospital stays and recovery times. These factors should be taken into consideration when evaluating the potential benefits and risks of this type of surgery (15). The microchannel is placed using the intermuscular approach to avoid scar tissue from previous surgery and minimize damage to muscle tissue. This technique can potentially decrease post-surgical pain, wound complications, and the risk of infection. To the best of our knowledge, this is the first report of bilateral microdecompression using a percutaneous microchannel unilateral approach for ASD 10 years after lumbar fusion. In this case, we analyzed clinicoradiologic data and made an accurate diagnosis before surgery. A surgical strategy was then developed to determine the extent of decompression segments and intraoperative decompression. The combination of percutaneous microchannel, microscope, burr and unilateral approach and bilateral decompression technology can fully decompress during operation and ensure the success of minimally invasive surgery.

In this study, we found that bilateral microdecompression via a unilateral approach under a microchannel is a safe and effective method for the treatment of ASD after lumbar fusion with good surgical outcomes. In the future, studies with longer follow-up and larger samples are needed to determine long-term prognosis. Furthermore, we have not yet conducted a comparative study of microdecompression and standard decompression. This is another limitation of this study and a new direction for future research.

## Conclusion

This report reports the successful treatment of a patient with ASD 10 years after lumbar fusion. Bilateral microdecompression via a unilateral approach under a microchannel is a safe and effective method for the treatment of ASD after lumbar fusion with good surgical outcomes.

## Data Availability

The original contributions presented in the study are included in the article/Supplementary Material, further inquiries can be directed to the corresponding authors.
